# CBD Supplementation Has a Positive Effect on the Activity of the Proteolytic System and Biochemical Markers of Honey Bees (*Apis mellifera*) in the Apiary

**DOI:** 10.3390/ani12182313

**Published:** 2022-09-06

**Authors:** Patrycja Skowronek, Łukasz Wójcik, Aneta Strachecka

**Affiliations:** Department of Invertebrate Ecophysiology and Experimental Biology, University of Life Sciences in Lublin, Akademicka 13, 20950 Lublin, Poland

**Keywords:** hemp extract, honey bee resistance, immunology, pollinators, cannabidiol, hemp oil, biochemistry, metabolism, biochemical pathway

## Abstract

**Simple Summary:**

The purpose of our study was to determine how CBD extract influences resistance in the hemolymph (insect blood) of honey bees in the hive test. The bees were divided into 3 groups: (CSy) bees fed with CBD in sugar syrup; (CSt) cotton strip with CBD placed in hive, (C) control bees fed sugar syrup. To determine the state of immunity, we used the analysis of the activity of the proteolytic system and biochemical markers, such as “liver tests”, and the concentration of selected ions and key compounds for the functioning of the organism. CBD extract increased the total protein concentration, proteases and their inhibitor activities in each age (except for acidic protease activities in the 21st and 28th day and alkaline protease inhibitor activities in the 28th day in the CSt group), increased concentrations of markers: ALP, AST, ALT; and glucose; triglycerides; cholesterol and creatinine. A decrease in concentration in experimental groups was noticed for urea acid and albumin compared to group C. Higher activities/concentrations of most of parameters were obtained in the CSy compared to the CSt and C. The CBD supplementation can positively influence bees’ resistance.

**Abstract:**

We examined how CBD extract influences the activity of the immune system in the hemolymph of honey bees in the hive test. The bees were divided into 3 groups: (CSy) bees fed with CBD in sugar syrup with glycerin; (CSt) cotton strip with CBD placed in hive bees fed pure sugar syrup, (C) control bees fed sugar syrup with glycerin. CBD extract increased the total protein concentrations, proteases and their inhibitor activities in each age (the except for acidic protease activities in the 21st and 28th day and alkaline protease inhibitor activities in the 28th day in CSt group) in comparison with group C. In the groups with the extract there was also an increase in the enzymatic marker activities: ALP, AST (decrease on day 28 for CSt), ALT; and non-enzymatic marker concentrations: glucose; triglycerides; cholesterol and creatinine. The urea acid and albumin concentrations were lower in CSy and CSt groups compared to the C group (higher concentration of albumin was displayed by control bees). Higher activities/concentrations of most of biochemical parameters were obtained in the CSy compared to the CSt and C. CBD supplementation can positively influence workers’ immune system.

## 1. Introduction

Nowadays, scientists report that we are witnessing a global ecological crisis [1]. This crisis concerns changes in the environment that affect not only people, but other living organisms, including those that, thanks to their unique biology, act as pillars stabilizing the ecosystem. An important biotic element is insects, which constitute one of the most diverse and numerous groups of animals. Among them we distinguish very useful insects, which additionally remain in close relationship with people. One of such insects is honey bees. Bees are responsible for pollination of approx. 70–80% of plants, the fruit/crops of which are part of the diet of humans and livestock [2]. Bees that have been kept in breeding since ancient times also provide products widely used in diet, pharmaceuticals and cosmetology, such as: honey, propolis, wax and royal jelly [3]. This confirms their inter-disciplinary role in the life cycle on Earth.

Recently, more and more scientific reports confirm the high decline in the population of honeybees as a result of climate change, environmental chemisation (pesticides), homogenization of agriculture (creation of monocultures, reduction of the diversity of bee food), human errors during bee breeding (treatment with bad agents, poor hygiene, adulteration wax) and other natural CCD (colony collapse disorder) etiological factors [4,5,6]. These above-mentioned factors caused a weakened immune system of pollinators and more susceptibility to the ingress of unwanted guests especially like: Nosema spp., *Varroa destructor* and other diseases such as rot (*Paenibacillus larvae*), viruses (deformed wings virus), etc. [4,7,8].

One of the key immune barriers responsible for dealing with pathogens is the proteolytic system, which consists of proteolytic enzymes (proteases) and protease inhibitors. Proteases are responsible for cutting the proteins of pathogens entering the bee’s organism. They are also responsible for digestion in lysosomes, activate proenzymes and pro-hormones, proliferative, fertile and apoptotic (cysteine proteases) and are involved in apoptosis, necrosis, virulence of microorganisms and digestion in lysosomes (metalloproteases) [9,10]. Protease inhibitors inhibit the action/protease activation of unwanted organisms [11,12]. Proteases and their inhibitors are found in one of the key tissues of bees: the hemolymph, which acts as the ‘blood’ of insects. The second key tissue related to immunity is the fat body, where most immune proteins are synthesized and the metabolic processes responsible for the proper functioning and development of insects take place. The fat body is the liver of insects. Proteins and other metabolites/substances/compounds from the fat body are transferred to the hemolymph where they are distributed throughout the body [13,14]. Therefore, laboratory ‘imaging’ of the hemolymph is one of the key indicators in determining the health of bees. In addition to the proteolytic system, in the hemolymph, we can examine the activity, concentrations of, e.g., enzymes such as alanine aminotransferase (ALT), aspartate (AST) and alkaline phosphatase (ALP), which reflect the functioning and damage of liver cells in bees of the fat body.

Due to reports of weakened bee immunity, many studies have focused on looking for agents that will naturally support these systems [15]. By enhancing the health of bees, they are expected to be able to deal with many factors at once. Recently, substances of natural (usually plant) origin, which can act as an immune biostimulator, have become very popular. Usually, the selected substances are characterized by a proven health-promoting effect, thanks to the content of active substances. In the case of bees, substances/compounds/products were tested, i.e., curcumin, vitamin C, caffeine, piperine, spirulina, coenzyme Q10, resveratrol, pollen substitutes, antiseptic herbal mixtures, yeast and even natural silage (as a source of *Lactobacillus*) [12,15,16,17,18,19,20,21,22]. Recently, we can also include hemp extracts (research carried out by our team) among the tested substances. Most of the mentioned biostimulants prolonged the life of bees by up to 33–38% (resveratrol, caffeine) [17,20]. In addition, some of the tested substances had a proven positive effect on the stimulation of the immune system by increasing the activity of the proteolytic system and the content of immune proteins (hemp, curcumin, coenzyme Q10) and antioxidant enzymes (coenzyme Q10, hemp, piperine, vitamin c) [12,15,16,18,23]. Coenzyme Q10 additionally increased the concentration of lipids in the body and of ions such as magnesium and calcium. Higher lipid levels were noted also after feeding bees with spirulina [19]. Caffeine, in addition, had a positive effect in the case of bees infected with *Nosema* spp. [17].

Despite the large range of tested substances, it has not been possible to find one effective agent that meets the needs of modern bee breeding and meets the strict requirements for the safety of its use. The gap can be filled with hemp, which has a strong antioxidant effect due to the content of active substances from the cannabinoid group [24,25,26]. The positive effects of hemp extracts have been described many times in relation to diseases such as depression, epilepsy, Alzheimer’s, appetite disorders, as an aid in the treatment of cancer and in multiple sclerosis [27,28,29,30,31]. There are also interesting studies in which the hemp extract helped to regenerate damaged brain tissues in rats, damaged as a result of the action of chemical agents [32]. Rats that took the extract showed faster regeneration than those that did not take the preparation. 

In the case of tests on invertebrates, in our previous studies we have shown that bees fed with hemp extract in a cage experiment showed a positive increase in immunity by stimulating the proteolytic and antioxidant system (growth recorded for all tested antioxidants) [23,26]. Taking into account our previous research and the results obtained showing the main effects of cannabidiol (CBD), for further testing we chose a commercial preparation containing the active ingredient CBD with a known concentration, which is one of the most described and tested active substances of cannabis along with THC (tetrahydrocannabinol) (THC has not been tested due to its psychoactive effect and legal conditions). We also added selected biochemical markers to the research in this paper, the results of which may help determine the effect of CBD on changes in the directions of metabolism in bees. The selected markers include: lipid compounds, i.e., cholesterol, triglycerides (the main compounds of the fat body), sugar: glucose (carbohydrate pathways and transformations take place in the fat body and affect the level of energy and hunger in bees), protein: albumin (transport of substances, buffer properties and anti-inflammatory effect), and metabolites of the end pathways, i.e., uric acid, creatinine (the concentration of metabolites gives information about the way the body is dealing with-metabolize the supplied supplements) [14,16,17]. Additionally, to get real results from the natural habitat of bees, we transferred our experiment from cage conditions to the beekeeping environment.

We assumed in the research that CBD extract has a positive effect on the immunity of honeybees by stimulating the proteolytic system and has a positive effect on the parameters of “liver” enzymes.

The aim of our study was to determine the activities of the proteolytic system, “liver” enzymes and additional metabolic biomarkers in bees supplemented with CBD extract under apiary conditions.

## 2. Materials and Methods

The research was carried out using 4-frame mating hives. Bees and queens were obtained from colonies of similar strength and age. Selected colonies were not treated preventively against *Varroa destructor* in order not to disturb the natural activity of the immune system. No *Nosema* spp. infection was detected in the colonies.

### 2.1. Preparatory Activities

#### 2.1.1. Getting Queens to Experience

Nine queens participated in the experiment (6 queens for the hive test + 3 queens to obtain 1-day-old workers). All 9 queens were sisters obtained from one source-queen. The young source-queen was caged within a queen-excluder comb-cage containing one empty comb in source-colony for egg laying. After 12 h, the source-queen was released. After 96 h, the larvae were transferred from the comb to the queen cup with the addition of mixed royal jelly in water. The future queens were placed in the queen-less colony for 7 days. Next, these queen pupae were transferred to the incubator (35 °C) for 7 days until emerged queens. Queen-sisters were placed in queen-less colonies until insemination. Nine queen-sisters were inseminated and 3 of them were placed in 3 colonies of similar strength (hives-Dadant Blatt; 20 frames; 435 × 150 mm), and 6 of them in mating hives.

#### 2.1.2. Preparation of Mating Hives

From the source-colony, fragments of combs containing different stages of larvae development were cut out and fit to the frames from the mating hives and placed in this hives. Additionally, worker bees of different ages (imago stages) were collected from the source-colony. Workers were divided, per 200 individuals to each of the mating hives. The queen was subjected to such constructed hives (containing all stages of development) (6 queens = 6 hives). During the week we checked whether the queen had been admitted, and within a month whether she had started lay eggs After a month of the colony’s functioning, 1-day-old bees were added to each of them, acquired from the 3 remaining queen-sisters in the colonies [33].

### 2.2. Obtaining and Marking of 1-Day-Old Bees

The each of three queen-sisters were caged within a queen excluder comb-cage containing one empty comb for egg laying for 12 h, in each of the three colonies, populating one-box hives (Dadant Blatt from Łysoń Beekeeping Company, Klecza Górna, Poland; frames: 435 × 150 mm^2^) [23]. The combs were marked and placed in their native colonies. After 20 days, these combs with broods were placed in an incubator (35 °C) that the 1-day-old bees emerged [23].

These 1-day-old workers were randomly marked with a colored oil marker depending on the assignment to the group. A total of 200 such workers were placed in each of the six mating hives. There were 2 mating hives per group (6 hives in total). The following groups were created: (1) CSy-experimental group, CBD in sugar syrup; (2) CSt-experimental group, CBD on a cotton strip; (3) C-control bees (supplemented with pure sugar syrup) [23,34].

### 2.3. Preparation and Administration of CBD Extract

We purchased a commercial hemp extract (HempOil) in the form of an oil with a concentration of 30% (3 g in 10 mL). The oil was obtained by means of CO_2_ extraction. CBD extract for group CSy was administered to the frameless chamber ad libitum in the 2nd, 4th and 6th days of the experiment. The oil was administered in a mixture with sugar syrup (1: 1 water with sugar) and glycerin in the ratio of 0.01:0.5:0.5 (extract: distilled water: glycerin). For the CSt group, the extract was given in a mixture with water and glycerin in a ratio of 0.8:1.5:1.5 (extract: distilled water: glycerin). Cotton strips measuring 2 by 10 cm were evenly moistened with the mixture and placed in the hives. The strip was wetted with the mixture in the 2nd, 4th and 6th days of the experiment.

### 2.4. Bees Sampling

From each of 6 colonies, we collected 10 marked bees once for week (10 bees × 6 colonies). Workers were collected on the following experiment days: 2, 7, 14, 21, 28 (CSt, CSy, C) and 35 (CSy). In the experiment, a total of 320 bees were collected (CSt-10 bees × 2 colonies × 5 samplings; CSy-10 bees × 2 colonies × 6 samplings; C-10 bees × 2 colonies × 5 samplings).

### 2.5. Collection of Hemolymph

The hemolymph was collected from each worker according to the methodology of Łoś and Strachecka (2018) [35]. A capillary (20 µL; ‘end to end’ type; without anticoagulant; Medlab Products, Raszyn, Poland) was individually inserted between the third and fourth tergite of living workers to obtain fresh hemolymph. Capillaries with the hemolymph of individual bees were placed in separate Eppendorf tubes with a capacity of 1.5 mL with a solution of 200 µL of 0.6% NaCl (10 bees = 10 tubes). Then, the material was frozen at −25 °C until biochemical analysis [35].

### 2.6. Biochemical Analyzes

#### 2.6.1. Proteolytic System and Total Protein Concentration

The proteolytic system activities and protein concentrations were determined in the hemolymph samples using the following methods:

Total protein concentration assay with the Lowry method modified by Schacterle and Pollack (1973) [36].

Acidic, neutral and alkaline protease activities according to the Anson method (1938) modified by Strachecka et al. (2011, 2012) [37].

Acidic, neutral and alkaline natural protease inhibitor activities according to the Lee and Lin method (1995) [38].

Details of these methods are provided in Łoś and Strachecka (2018) manuscript [35].

#### 2.6.2. Metabolic Markers

In the hemolymph samples, the following biomarkers were determined using a commercial kit with modified instructions by Łoś and Strachecka (2018) [35]:

1. Enzymatic biomarkers activities, i.e., alkaline phosphatase (ALP), aspartate transaminase (AST), alanine aminotransferase (ALT);
2. Energy reserves, i.e., glucose, triacyloglycerol and cholesterol concentrations;
3. Creatinine concentration;
4. Uric acid concentration; and
5. Albumin concentration

### 2.7. Statistical Analysis

The results were analyzed using Statistica formulas, version 13.3 (2017) for Windows, StatSoft Inc., Tusla, OK, USA. The mixed-model two-way ANOVA followed by post hoc Tukey HSD tests (*p *=  0.05) was used to compare the results for each basic immunity system parameter (total protein concentration, protease activities, protease inhibitor activities, biomarker activities/concentrations) of honey bee workers depending on the method of administration (hemp on strip and hemp in sugar syrup) and the day (2, 7, 14, 21, 28, 35 day) of supplementation with hemp extract.

## 3. Results

In most cases, groups with bees supplemented with CBD extract had higher concentrations and activities of immune parameters.

### 3.1. The Total Protein Concentration

**Figure 1:**
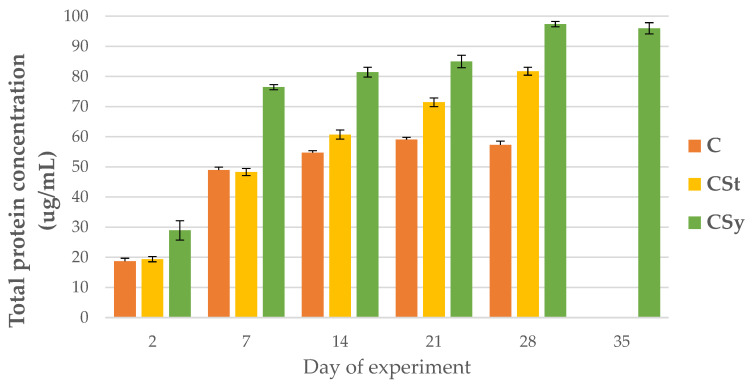
Total protein concentration in the 2, 7, 14, 21, 28, 35-day-old workers’ hemolymph after two methods of supplementation with hemp extract: C-control (pure sugar syrup), CSy-hemp extract in syrup, CSt-hemp extract on strips. Two-Way ANOVA: supplementation method F_(2, 156)_ = 31,397; *p* = 0.0000, se ± 2.931476–2.649163; days of supplementation: F_(4, 143)_ = 6677.0, *p* = 0.0000, se ± 0.268945–0.465826; supplementation method × days of supplementation F_(8, 143)_ = 203.83, *p* = 0.0000, se ± 0.465826–0.491024.

The total protein concentrations increased with workers’ age for all groups. The protein concentration was always higher in the CSt group compared to the CSy and C groups. The lowest protein concentrations were observed in C group in all samplings, except on the seventh day, when the lowest value was in the CSt group (Figure 1).

### 3.2. The Proteolytic System Activities

**Figure 2:**
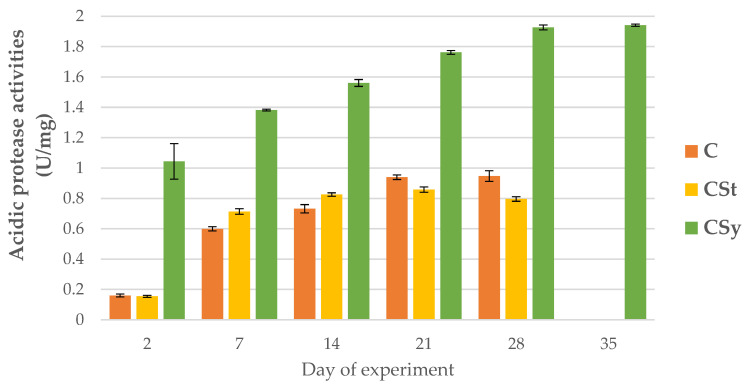
Acidic protease activities in the 2, 7, 14, 21, 28, 35-day-old honey bees’ hemolymph after two methods of supplementation with hemp extract: C-control (pure sugar syrup), CSy-hemp extract in syrup, CSt-hemp extract on strips. Two-Way ANOVA: supplementation method: F_(2, 156)_ = 182,90, *p* = 0.0000, se ± 0.042535–0.038439; days of supplementation: F_(4, 143)_ = 2518.9, *p* = 0.0000, se ± 0.006184–0.01071; supplementation method × days of supplementation: F_(8, 143)_ = 71.420, *p* = 0.0000, se ± 0.01071–0.01129.

**Figure 3:**
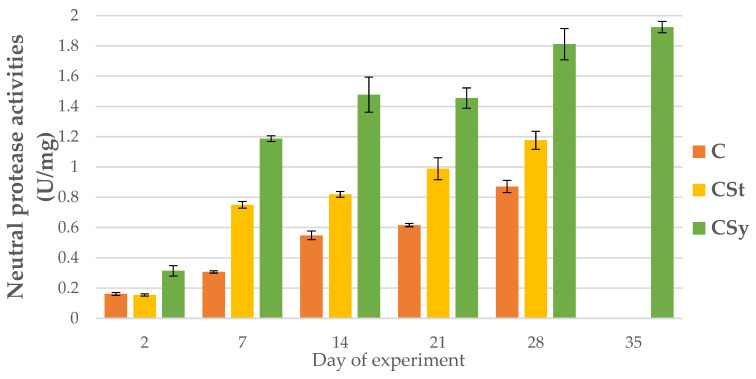
Neutral protease activities in the 2, 7, 14, 21, 28, 35-day-old honey bees’ hemolymph after two methods of supplementation with hemp extract: C-control (pure sugar syrup), CSy-hemp extract in syrup, CSt-hemp extract on strips (two-Way ANOVA) supplementation method: F_(2, 156)_ = 64.957, *p* = 0.0000, se ± 1.361555–0.500698 days of supplementation: F_(4, 143)_ = 1754.7, *p* = 0.0000, se ± 0.009558–0.016555; supplementation method × days of supplementation F_(8, 143)_ = 110.82, *p* = 0.0000, se ± 0.016555.

**Figure 4:**
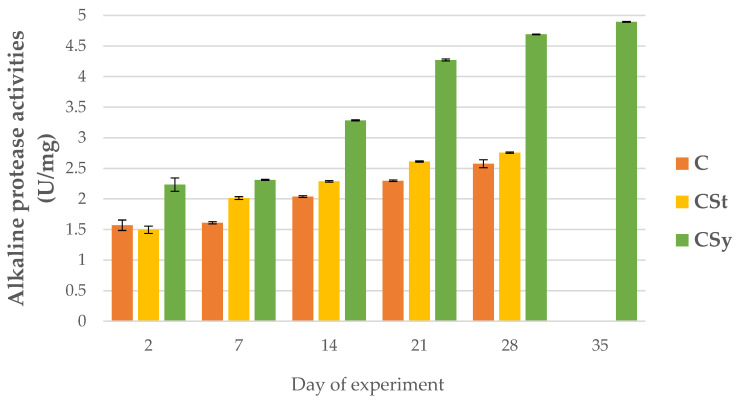
Alkaline protease activities in the 2, 7, 14, 21, 28, 35-day-old honey bees’ hemolymph after two methods of supplementation with hemp extract: C-control (pure sugar syrup), CSy-hemp extract in syrup, CSt-hemp extract on strips; two-Way ANOVA: supplementation method: F_(2, 156)_ = 75.790, *p* = 0.0000, se ± 0.105637–0.096433; days of supplementation: F_(4, 143)_ = 7364.6, *p* = 0.0000, se ± 0.007821–0.013546; supplementation method × days of supplementation F_(8, 143)_ = 856.83, *p* = 0.00094, se ± 0.013546.

**Figure 5:**
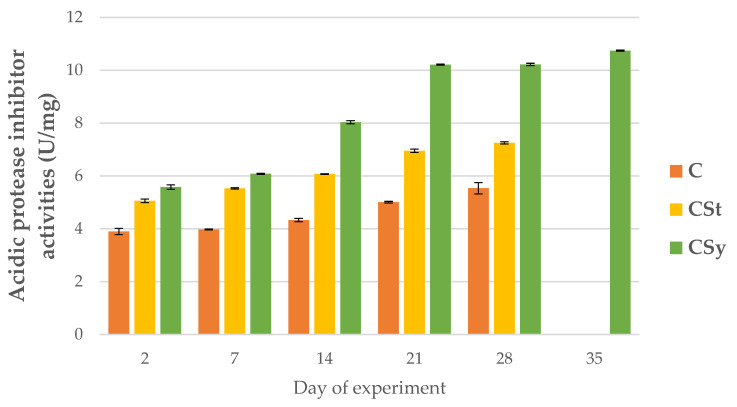
Acidic protease inhibitor activities in the 2, 7, 14, 21, 28, 35-day-old honey bees’ hemolymph after two methods of supplementation with hemp extract: C-control (pure sugar syrup), CSy-hemp extract in syrup, CSt-hemp extract on strips. (Two-Way ANOVA: supplementation method: F_(2, 156)_ = 108.68, *p* = 0.0000, se ± 0.201107–0.181740; days of supplementation: F_(4, 143)_ = 8449.9, *p* = 0.0000, se ± 0.013698–0.023726; supplementation method × days of supplementation F_(8, 143)_ = 1230.1, *p* = 0.0000, se ± 0.023726–0.025009.

**Figure 6:**
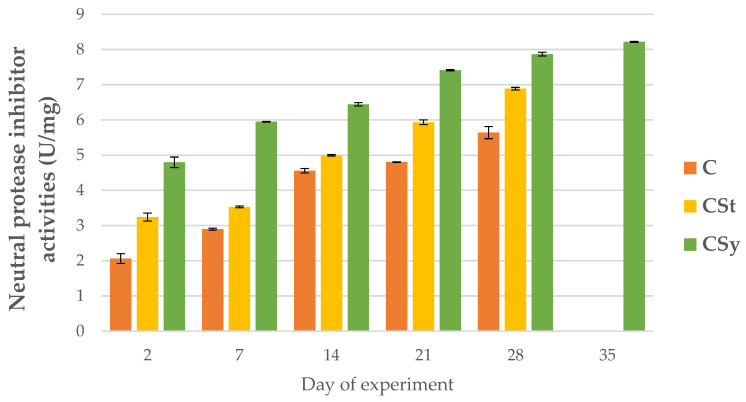
Neutral protease inhibitor activities in the 2, 7, 14, 21, 28, 35-day-old honey bees’ hemolymph after two methods of supplementation with hemp extract: C-control (pure sugar syrup), CSy-hemp extract in syrup, CSt-hemp extract on strips. (Two-Way ANOVA: supplementation method: F_(2, 156)_ = 66.832, *p* = 0.0000, se ± 0.185759–0.167870; days of supplementation: F_(4, 143)_ = 8999.5, *p* = 0.0000, se ± 0.014626–0.025333; supplementation method × days of supplementation F_(8, 143)_ = 156.29, *p* = 0.0000, se ± 0.025333.

**Figure 7:**
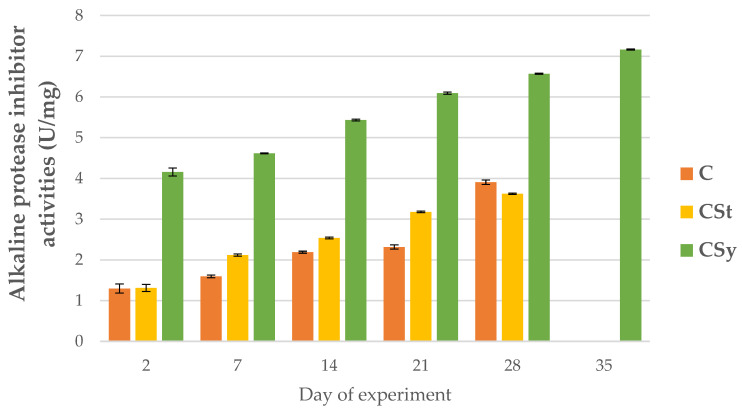
Alkaline protease inhibitor activities in the 2, 7, 14, 21, 28, 35-day-old honey bees’ hemolymph after two methods of supplementation with hemp extract: C–control (pure sugar syrup), CSy–hemp extract in syrup, CSt–hemp extract on strips. (Two-Way ANOVA: supplementation method: F_(2, 156)_ = 225.68, *p* = 0.0000, se ± 0.121993–0.133636; days of supplementation: F_(4, 143)_ = 10359, *p* = 0.0000, se ± 0.009407–0.016; supplementation method × days of supplementation F_(8, 143)_ = 266.45, *p* = 0.0000, se ± 0.016–0.016866.

The addition of CBD to the syrup (CSy group) caused an unexpected increase in the activities of proteases and their inhibitors compared to the other two groups (CSt and C; Figure 2, Figure 3, Figure 4, Figure 5, Figure 6 and Figure 7). These activities increased with the age of the workers in all groups, with the exception of acid proteases in C and CSt groups, in which decreased activities were noted in old bees (Figure 2).

### 3.3. The Enzymatic Biomarkers

**Figure 8:**
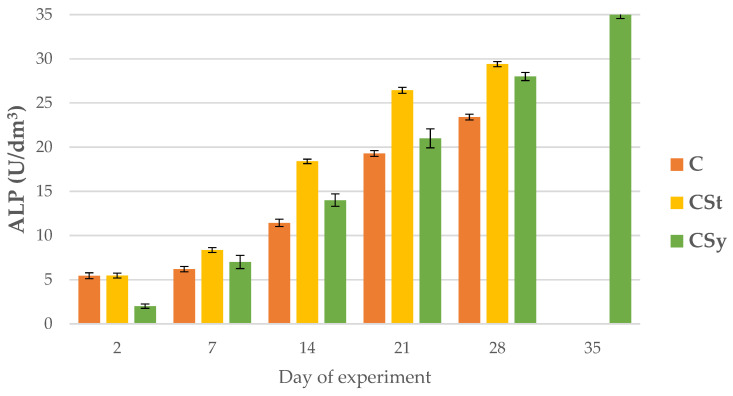
ALP activities in the 2, 7, 14, 21, 28, 35-day-old honey bees’ hemolymph after two methods of supplementation with hemp extract: C-control (pure sugar syrup), CSy-hemp extract in syrup, CSt-hemp extract on strips. (Two-Way ANOVA: supplementation method: F_(2, 156)_ =18.598, *p* = 0.0000 se ± 1.275927–1.411898; days of supplementation: F_(4, 143)_ = 13672, *p* = 0.0000, se ± 0.089307–0.151897; supplementation method × days of supplementation F_(8, 143)_ = 300.67, *p* = 0.0000, se ± 0.0151897.

**Figure 9:**
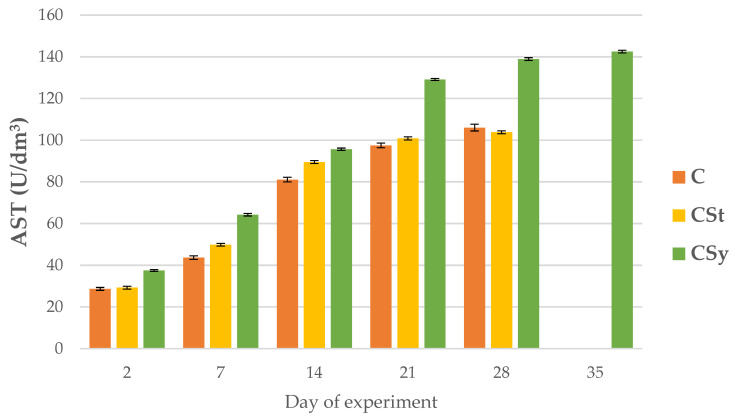
AST activities in the 2, 7, 14, 21, 28, 35-day-old honey bees’ hemolymph after two methods of supplementation with hemp extract: C-control (pure sugar syrup), CSy-hemp extract in syrup, CSt-hemp extract on strips. Two-Way ANOVA: supplementation method: F_(2, 156)_ = 13.130, *p* = 0.00001, se ± 4.891108–4.420075; days of supplementation: F_(4, 143)_ = 58610, *p* = 0.0000; se ± 0.149875–0.259592; supplementation method × days of supplementation F_(8, 143)_ = 705.49, *p* = 0.0000, se ± 0.2595952–0.273634.

**Figure 10:**
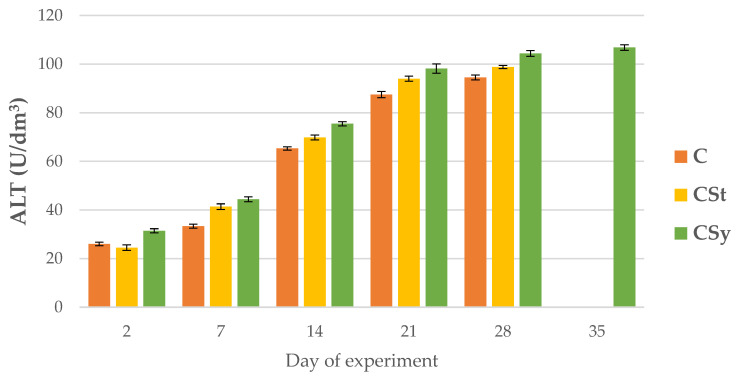
ALT activities in the 2, 7, 14, 21, 28, 35-day-old honey bees’ hemolymph after two methods of supplementation with hemp extract: C-control (pure sugar syrup), CSy-hemp extract in syrup, CSt-hemp extract on strips. Two-Way ANOVA: supplementation method: F_(2, 156)_ = 4.3070, *p* = 0.01511, se ± 3.750347–4.150009; days of supplementation: F_(4, 143)_ = 26349, *p* = 0.0000, se ± 0.19505–0.337837; supplementation method × days of supplementation F_(8, 143)_ = 30.235, *p* = 0.0000, se ± 0.337837.

ALP, AST and ALT activities increased with age of the workers in all group (Figure 8, Figure 9 and Figure 10).

Regardless of the method of administration (syrup or strips; CSy or CSt), CBD increased the ALP activities in the hemolymph of workers of all ages compared to the control (C) group. Between 7 and 28 days of workers’ age, higher ALP activities were observed in the CSt group compared to CSy and C (Figure 8).

AST and ALT activities were higher in groups administrated with CBD compared to C group; with the highest values always being observed in the CSy group (Figure 9 and Figure 10). AST activities in the 28-day-old workers from the CSt group were lower compared to the CSy and C groups (Figure 9).

### 3.4. The Non-Enzymatic Biomarkers (Including Energy Reserves)

**Figure 11:**
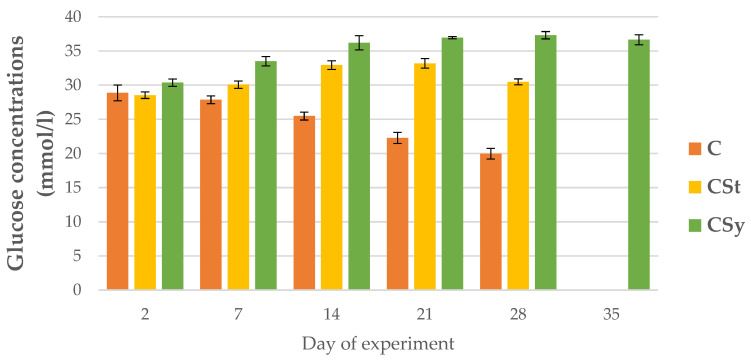
Glucose concentrations in the 2, 7, 14, 21, 28, 35-day-old workers’ hemolymph after two methods of supplementation with hemp extract: C-control (pure sugar syrup), CSy-hemp extract in syrup, CSt-hemp extract on strips. Two-Way ANOVA: supplementation method: F_(2, 156)_ = 195.30, *p* = 0.0000, se ± 0.388458–0.351048; days of supplementation: F_(4, 143)_ = 63.193, *p* = 0.0000, se ± 0.217085–0.125334; supplementation method × days of supplementation F_(8, 143)_ = 249.21, *p* = 0.0000, se ± 0.217085–0.228828.

The glucose concentrations in the hemolymph of the control bees decreased over the course of their lives, while in the groups supplemented with CBD they increased with the days. In the CSt group, a decrease in concentration was noted on day 28. The experimental groups showed higher glucose levels than the control. The highest glucose concentrations were in the CSy group (Figure 11).

**Figure 12:**
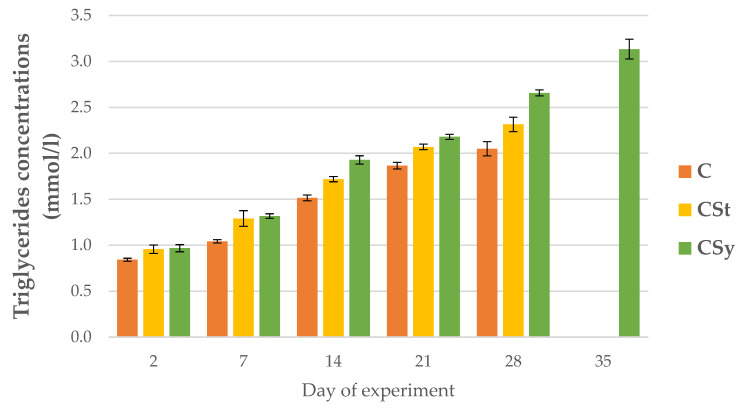
Triglyceride concentrations in the 2, 7, 14, 21, 28, 35-day-old workers’ hemolymph after two methods of supplementation with hemp extract: C-control (pure sugar syrup), CSy-hemp extract in syrup, CSt-hemp extract on strips. Two-Way ANOVA: supplementation method: F_(2, 156)_ = 12.927, *p* = 0.00001, se ± 0.085425–0.077199; days of supplementation: F_(4, 143)_ = 3683.5, *p* = 0.0000, se ± 0.009509–0.016471; supplementation method × days of supplementation F_(8, 143)_ = 33.426, *p* = 0.0000, se ± 0.016471–0.017362.

The triglyceride concentrations were higher in the groups supplemented with cannabis compared to the control group. The concentration increased with the age of the bees in all groups. The highest concentrations were recorded for the CSy group (Figure 12).

**Figure 13:**
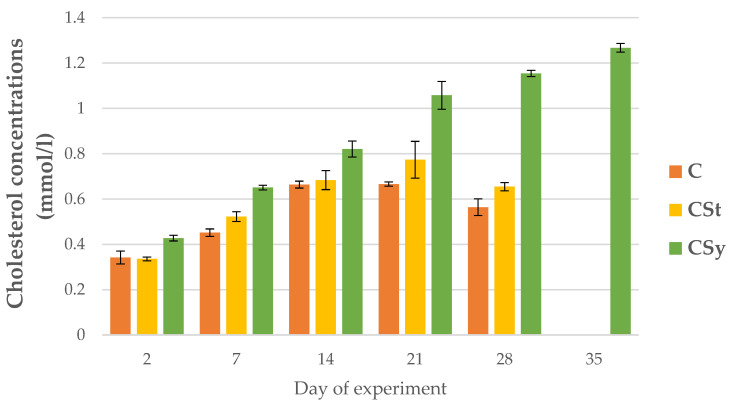
Cholesterol concentrations in 2, 7, 14, 21, 28, 35-day-old workers’ hemolymph after two methods of supplementation with hemp extract: C-control (pure sugar syrup), Csy-hemp extract in syrup, Cst-hemp extract on strips. Two-Way ANOVA: supplementation method: F_(2, 156)_ = 44.905, *p* = 0.0000 se ± 0.027850–0.030509; days of supplementation: F_(4, 143)_ = 997.34, *p* = 0.0000, s se ± 0.006106–0.010575; supplementation method × days of supplementation F_(8, 143)_ = 107.76, *p* = 0.0000, se ± 0.010575–0.11147.

A continuous increase in cholesterol concentrations was observed with the age of workers in the CSy group (Figure 13). In this group, the highest cholesterol concentrations were in relation to the other groups. For CSt and C groups, the increase in concentration continued until day 21, on day 28, the values decreased. The CSt group, despite a similar downward trend on day 28, had a higher cholesterol concentration than the C group (Figure 13).

**Figure 14:**
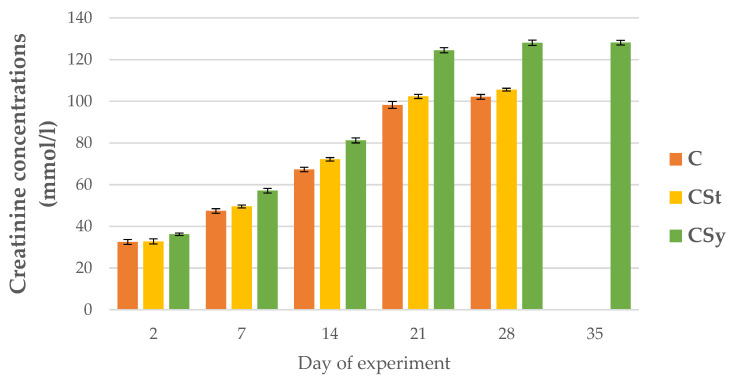
Creatinine concentrations in the 2, 7, 14, 21, 28, 35-day-old workers’ hemolymph after two methods of supplementation with hemp extract: C-control (pure sugar syrup), CSy-hemp extract in syrup, CSt-hemp extract on strips. Two-Way ANOVA: supplementation method: F_(2, 156)_ = 8.8603, *p* = 0.00023, se ± 4.553192–4.114702; days of supplementation: F_(4, 143)_ = 29106, *p* = 0.0000, se ± 0.200493–0.347264; supplementation method × days of supplementation F_(8, 143)_ = 248.02, *p* = 0.0000, se ± 0.347264–0.366048.

The creatinine concentrations were always higher in groups administrated CBD than C group and these values were the highest in the hemolymph workers in the CSy group. The creatinine concentrations increased with the age of the bees for all groups (Figure 14).

**Figure 15:**
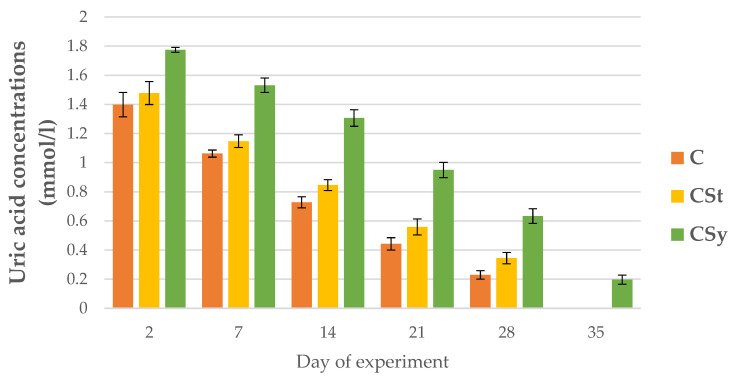
Uric acid concentrations in the 2, 7, 14, 21, 28, 35-day-old workers’ hemolymph after two methods of supplementation with hemp extract: C-control (pure sugar syrup), CSy-hemp extract in syrup, CSt-hemp extract on strips. Two-Way ANOVA: supplementation method: F_(2, 156)_ = 5.4955, *p* = 0.00494, se ± 0.067071–0.060612; days of supplementation: F_(4, 143)_ = 2605.7, *p* = 0.0000, se ± 0.008902–0.015419; supplementation method × days of supplementation F_(8, 143)_ = 8.4650, *p* = 0.0000, se ± 0.015419–0.016253.

**Figure 16:**
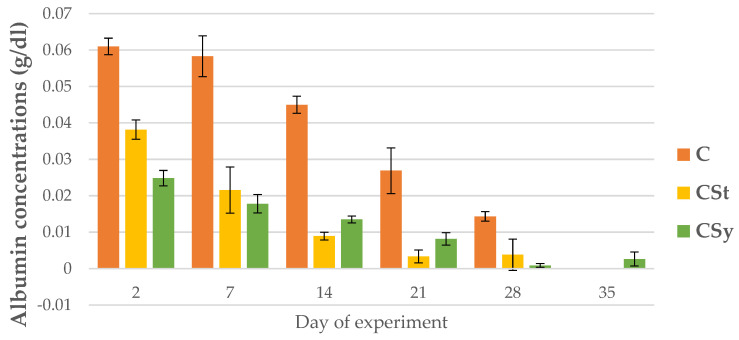
Albumin concentration in the 2, 7, 14, 21, 28, 35-day-old workers’ hemolymph after two methods of supplementation with hemp extract: C-control (pure sugar syrup), CSy-hemp extract in syrup, CSt-hemp extract on strips. Two-Way ANOVA: supplementation method: F_(2, 156)_ = 69.318, *p* = 0.0000, se ± 0.002002–0.011302; days of supplementation: F_(4, 143)_ = 561.33, *p* = 0.0000, se 0.000596–0.001033; supplementation method × days of supplementation F_(8, 143)_ = 47.417, *p* = 0.0000, se 0.001033–0.001089.

The uric acid and albumin concentrations decreased with the aging of bees for all groups (Figure 15 and Figure 16).

The addition of CBD (regardless of administration-syrup or strips; CSy or CSt) caused an increase in the uric acid concentrations in each day of life of workers in comparison with group C (Figure 15). The CSy group had the highest uric acid concentrations on each analysis day.

The albumin concentrations were the highest in the bees’ hemolymph from the C group on each sampling day. By day 7, the CSt group had higher the albumin concentrations, however, from day 14, the albumin concentrations were higher in CSy compared to CSt (Figure 16).

## 4. Discussion

Pure CBD extract as a potent active substance significantly influenced the changes in the parameters of the immune system in our study. During the experiment, two methods of administering the supplement were used. As this research shows, the method of administration has a significant impact on changes and their intensity in the activities of the proteolytic system and the concentrations of biochemical markers. The intensity of the effect is also reflected in the lifespan of the bees. The bees from the CSy group showed the highest parameters of activity and were the only ones to survive until the 35th day of the experiment. The CSt group, despite the higher activity than in the C group, lived until the 28th day.

The results of total protein concentration obtained in this experiment confirm the results obtained in the study by Skowronek et al. (2021) which contain results only from cage experiment on 7 and 14-days-old bees [23]. The higher concentration of total protein in the groups supplemented with CBD indicate a higher production of immune proteins in the bees’ organism. Regardless of the method of administration, CBD entered the bees’ digestive system, where from the gut it could be further absorbed by other surrounding tissues and affect the production of proteins in the fat body. The lower concentration of proteins in the CSt group could be caused by a delayed action due to the longer route of the extract entering the gastrointestinal tract (not direct administration). The same tendency is visible for other parameters characterizing the humoral immunity of bees. Supplementation could increase the concentration of Ca^2+^ ions, which affect the functioning of many enzymes involved in the body’s defense [39]. The activity of specific calcium phospholipases (cPLA2-Ca^+2^-dependent cytosolic phospholipase (A2), mentioned in Skowronek et al. (2022)), depends on the calcium content [26]. They influence the production of eicosanoids, which support immune processes at the level of the humoral response of the fat body [40]. The use of pure CBD and the achievement of a comparable effect with the studies carried out earlier in cages confirms that it is this substance that is responsible for the effects obtained in the previous experiment. This also confirms the similar effect of lipophilic compounds in supplementation on bees, i.e., CBD or coenzyme Q10 [12].

For all proteases and protease inhibitors, we noted higher activities of these parameters for the CSy group. In the CSt group, the exceptions were acid proteases and alkaline inhibitors. Contrary to the cage studies, proteases could be activated and increased because the bees kept in natural conditions were not exposed to the negative/stress factor like the cage. The cage is a foreign environment (without queen) for bees and limits the functioning in line with their physiology. Under natural conditions, bees may be exposed to biotic or abiotic stress, which is visible, for example, in the activity of serine proteases. In this case, the increase in the activity of the proteolytic system of the supplemented bees may be associated with a stronger and faster response to possible negative factors and thus their faster neutralization. In the case of the cages, the bees were isolated from these factors.

A positive effect on immunogenic tissues, in particular on the fat body, can be observed in higher activities of markers such as ALP, AST and ALT. As we have described before, and as confirmed by many other publications, the fat body functions as the liver of bees. However, as it turns out, in invertebrates, the increase in the concentration of these substances is not associated with damage to the cells of the fat body. The increase in ALP, AST and ALT was noted with other supplements defined as positive, i.e., caffeine, coenzyme Q10, curcumin [12,16,17]. After the collection of previously published articles, we conclude that these parameters can be used to determine the health level of bees like in humans. However, in the case of bees, the increase in these parameters is a positive effect (not inflammation as in the case of the research’s interpretation of humans). In the case of ALP, we observed a higher concentration in the CSt group. This may be related to the sealing of physiological barriers and the deposition of evaporating CBD on the bee’s cuticle, as well as its penetration and reaching the fat body. However, this effect only occurred with this one enzyme. In order to establish this relationship, more research and knowledge of the biochemical functioning of the fat body should be obtained. In the studies published so far, it turns out that the reduced values of the activities of these enzymes were noted with the negative impact of administering amphotericin B, bromfenvinfos and formic acid to bees, which had an immunosuppressive effect, which confirms that the decrease in these parameters proves the poor health of bees [41,42]. In addition to the increase in liver enzymes, all supplemented groups had a high concentration of triglycerides and cholesterol, which are the main fatty compounds in the fat body (over 50%) [14]. Triglycerides and cholesterol are the main equivalent of reserve substances and together form fat droplets embedded in the cells. When needed, triglycerides are converted by metabolic pathways into diglycerides or fatty acids and transported to tissues when needed. The increase in triglycerides suggests again the previously indicated effect of CBD on the fat body by increasing the concentration of Ca^2+^ ions [43]. The level and homeostasis of these ions has a large impact on the activation and course of pathways (lipolysis, lipogenesis) and the regulation of the level of lipid reserves [13,14,44]. In addition, calcium ions by regulating lipid metabolism also affect the development and aging of the body (diapause, metamorphosis). Similar trends occurred in the case of using other supplements in the publications of the Strachecka team [12,16,17].

A higher level of glucose indicates a high availability of sugars in the diet (no hunger), thanks to which the bee can carry out all the necessary metabolic processes related to the use of high-energy organic compounds. Additionally, a high level of glucose in the organism of bees may have a positive effect on the level of energy needed for flights and for obtaining food. Glucose is a key carbohydrate that is both a basic substrate and a product of metabolic pathways. Furthermore, trehalose is synthesized from glucose, which affects the lipid metabolism by inactivating lipase enzymes [14,45]. The positive effect of increased glucose concentration in the hemolymph may also confirm that bees that are infected with *Nosema* spp. or *Varroa destructor* parasites show a lower glucose concentration, as a result of which bees experience energy stress [46]. These results are inconsistent with the results obtained from the cage studies, but it is also due to a different energy balance in the bees remaining in the cages, restricting their movement, and in the hives where the bees have access to honeyflow and at the same time show different energy consumption (flights).

The increase in the concentration of creatinine, albumin and urea acid indicates that, despite a different chemical structure, CBD was metabolized similarly to curcumin and coenzyme Q10, which are referred to together with CBD as antioxidants or substances with antioxidant potential [12,16]. In the case of bees from the CSt group, they showed a lower concentration of creatinine and uric acid due to the fact that supplementation was not directly consumed in such amounts as in the CSy group, which could affect the natural metabolism. It should be noted that although the groups where the bees consumed the extract had higher values, the trend with the age of the bees was the same as for the control bees.

## 5. Conclusions

CBD extract may prove to be a good supplement and can have positive effect on the immune system of honeybees by stimulating the proteolytic system and other metabolic parameters. We observed a positive effect in our study with two methods of administration, but the results show that supplementation in sugar syrup allows for higher immunity parameters in relation to the administration of CBD on strips in our experiment. However, both methods can be used due to a possible increase in potential of humoral immunity and anatomical and physiological barriers in honey bees.

## Data Availability

The datasets and materials for this study results that have been used, analyzed and presented in this manuscript are not publicly available. Available on University of Life Sciences in Lublin. At the justified request of the interested party, they may be made available by the respective author.
